# Deciphering the Heterogeneity of Vibration Therapy for Post-Stroke Gait: A Systematic Review and Meta-Regression Protocol

**DOI:** 10.3390/biomedicines14092004

**Published:** 2026-09-06

**Authors:** Jeong-Woo Seo, Jin Mi Chun, Se-Ra Park, Ji-Woo Seok, Jung-Dae Kim

**Affiliations:** 1 Digital Health Research Division, Korea Institute of Oriental Medicine, Daejeon 34054, Republic of Korea; 2 Korean Medicine Convergence Research Division, Korea Institute of Oriental Medicine, Daejeon 34054, Republic of Korea

**Keywords:** stroke, vibration therapy, whole-body vibration, gait rehabilitation, systematic review, meta-regression

## Abstract

**Background**: Despite the increasing use of vibration therapy (VT) in neurorehabilitation, its efficacy on post-stroke gait recovery remains controversial due to substantial heterogeneity in study protocols and outcome assessments. Previous systematic reviews have often aggregated disparate gait metrics and stroke stages, potentially obscuring domain-specific therapeutic effects and limiting clinical applicability. Furthermore, the specific influence of vibration parameters, such as frequency and amplitude, on gait outcomes has not been sufficiently quantified. **Objective**: This protocol outlines a systematic review and meta-regression analysis designed to rigorously evaluate the clinical efficacy of VT on the multidimensional recovery of gait function in stroke survivors. Unlike prior reviews, this study aims to decipher the heterogeneity of treatment effects by analyzing outcome variations based on specific gait domains and exploring associations between individual vibration parameters and treatment effect estimates. **Methods**: In adherence to PRISMA-P guidelines, a comprehensive search will be conducted across PubMed, Embase, Cochrane Library, Web of Science, and Scopus for randomized and non-randomized controlled trials involving adult stroke survivors. Interventions will include whole-body vibration (WBV) and focal muscle vibration (FMV) compared to sham or standard care. To address the multidimensional nature of gait, outcomes will be strictly categorized into four domains: (1) gait speed, (2) gait endurance, (3) functional mobility, and (4) spatiotemporal parameters. Methodological quality will be assessed using the Cochrane Risk of Bias 2 (RoB 2) and Risk of Bias In Non-randomized Studies of Interventions (ROBINS-I) tools. Data synthesis will utilize random-effects models, and prespecified meta-regression analyses will be employed to investigate the influence of key moderators, including vibration frequency, amplitude, and session duration. **Discussion**: This review aims to move beyond simple efficacy verification by exploring potential moderators of treatment effects and generating hypotheses regarding vibration parameters that may be associated with more favorable gait outcomes. By providing granular evidence across distinct gait domains and analyzing parameter-specific effects, this study is expected to resolve existing conflicting evidence and offer precise guidance for the clinical application of VT in post-stroke gait rehabilitation.

## 1. Background

Stroke remains a leading cause of adult disability globally, with over 70% of survivors experiencing persistent motor impairments and gait dysfunction [1,2,3]. These deficits, characterized by gait asymmetry and reduced walking speed, significantly restrict independent activities of daily living and quality of life [2,3]. Importantly, post-stroke gait impairment reflects not only muscle weakness but also impaired sensorimotor integration and altered postural control strategies, which together compromise safe and efficient locomotion [2,3]. In the realm of neurorehabilitation, non-invasive sensory stimulation has gained prominence as a strategy to induce neuroplasticity without the risks associated with pharmacological or surgical interventions [4,5]. Such approaches aim to enhance afferent input to the central nervous system and facilitate motor relearning through repeated sensorimotor engagement [4].

Among these modalities, vibration therapy (VT), encompassing both whole-body vibration (WBV) and focal muscle vibration (FMV), is hypothesized to enhance motor output by stimulating muscle spindles and Ia afferents, thereby modulating spinal reflex excitability and increasing corticospinal drive [5,6,7]. Through oscillatory mechanical stimulation, VT may evoke tonic vibration reflexes and alter neuromuscular activation patterns, potentially supporting functional motor recovery after stroke [5,6].

Consequently, VT is widely utilized in clinical settings, and a substantial volume of randomized controlled trials (RCTs) has investigated its impact on functional recovery [7,8,9]. Numerous studies report that VT significantly mitigates spasticity, increases lower limb muscle strength, and improves balance control [8,9]. These effects are considered clinically relevant, as reduced spasticity and improved postural stability are prerequisites for effective gait performance. Specifically, regarding gait, recent evidence suggests VT can facilitate the sensory integration necessary for locomotion, with some trials reporting significant improvements in step length and walking speed [10,11,12]. For example, beneficial effects have been observed in functional mobility tasks when vibration is combined with visual control training or conventional physical therapy, indicating a potential synergistic effect between VT and task-oriented rehabilitation [11,12].

However, despite the abundance of clinical trials, the consensus regarding the efficacy of VT on gait performance remains elusive due to conflicting quantitative evidence [13,14,15,16,17]. While some studies report benefits, others indicate negligible or non-significant effects, particularly in chronic stroke populations or when VT is applied as a stand-alone intervention [13,14,15,16,18]. For instance, a systematic review by Yang et al. found that while VT showed potential for balance improvement, its effect on mobility was not statistically significant, yielding a standardized mean difference (SMD) of 0.45 (95% CI: −0.46 to 1.37) [14]. Similarly, Lu et al. (2015) reported a weighted mean difference of −50.40 m for the 6-Minute Walk Test (6MWT), suggesting no significant improvement in gait endurance compared to control interventions [15]. Furthermore, a comprehensive meta-analysis by Park et al. (2018) revealed that while VT had a large effect size for spasticity (1.24), the aggregated effect size for gait function was trivial (0.09), raising questions about its clinical utility for locomotor rehabilitation [17].

This discrepancy in outcomes is largely attributed to high heterogeneity across studies, driven by variations in vibration parameters (e.g., frequency and amplitude) and intervention protocols (e.g., dosage, posture, and session duration) [17,19,20]. Yang and Butler (2020) noted that although controlled WBV yielded a small but statistically significant immediate effect on mobility (SMD = 0.34), the long-term retention effects remained unclear, emphasizing the critical role of dosing parameters and intervention design [20]. Additionally, emerging evidence suggests that the physiological and functional responses to vibration are frequency-dependent, with low and high frequencies potentially eliciting distinct effects on neuromuscular activation, balance, and gait-related outcomes [19,20,21].

Previous systematic reviews have attempted to synthesize these findings but face significant methodological limitations [14,15,17,19]. Many reviews have aggregated heterogeneous stroke stages (acute, subacute, and chronic) or pooled disparate outcome measures, such as static balance scales and dynamic gait speed, thereby diluting domain-specific therapeutic effects [14,15,17]. Crucially, gait is a multidimensional construct comprising speed, endurance, and complex spatiotemporal characteristics; however, prior analyses often lack a granular categorization of these domains, limiting the interpretability of pooled estimates [22,23].

To address these gaps and resolve conflicting evidence, this study outlines a protocol for a comprehensive systematic review and meta-analysis with a strategic focus on multidimensional gait assessment following VT intervention. Adopting methodologies from recent rigorous reviews [19,22,23,24], gait outcomes will be categorized into distinct domains: (1) gait speed (e.g., 10-Meter Walk Test (10MWT)), (2) gait endurance (e.g., 6MWT), (3) spatiotemporal parameters (e.g., cadence and stride length), and (4) functional mobility (e.g., Timed Up and Go (TUG)). Furthermore, meta-regression analyses will be employed to explore associations between key vibration parameters, specifically frequency and amplitude, and variations in treatment effect estimates. This approach aims to move beyond simple efficacy verification and provide precise, evidence-based guidance for the application of VT in post-stroke gait rehabilitation [19,20,21,24].

## 2. Methods/Design

### 2.1. Protocol and Registration

This systematic review and meta-analysis protocol has been conceptually designed in strict adherence to the Preferred Reporting Items for Systematic Review and Meta-Analysis Protocols (PRISMA-P) guidelines [25]. The primary objective of this review is to rigorously evaluate the clinical efficacy of VT on the multidimensional recovery of gait function in stroke survivors. Unlike previous reviews that have often aggregated disparate gait metrics, this study aims to dissect the heterogeneity of treatment effects by quantitatively analyzing outcome variations based on stimulation parameters and specific gait domains. The protocol was registered in the International Prospective Register of Systematic Reviews (PROSPERO; CRD420251110025) on 23 July 2025 to ensure transparency and prevent duplication of efforts. At the time of registration, formal searching and study screening had commenced, whereas data extraction had not yet begun. Any substantial amendments made to the protocol during the review process will be documented in the final manuscript with a clear rationale.

### 2.2. Population, Intervention, Comparator, Outcome (PICO) Question

To address the complexities of post-stroke gait recovery, this review adopts a structured PICO framework. The Population will consist of adult participants (aged 18 years or older) with a clinically confirmed diagnosis of ischemic or hemorrhagic stroke, across all stages of post-stroke recovery. The Intervention of interest is mechanical VT, including both WBV and FMV, administered either as an isolated intervention or as an adjunct to conventional rehabilitation. Although both modalities are included within the scope of mechanical VT, WBV and FMV will be treated as distinct intervention categories in the quantitative synthesis because of differences in stimulation site, mode of delivery, and biomechanical transmission. The Comparator will include control conditions such as sham vibration (placebo), no treatment, or standard care (e.g., conventional physical therapy) to isolate the specific physiological effects of the vibratory stimulus. The Outcomes will be strictly categorized into four distinct domains of gait performance to facilitate granular analysis: (1) Gait Speed, (2) Gait Endurance, (3) Functional Mobility, and (4) Spatiotemporal Parameters.

### 2.3. Eligibility Criteria

We will include RCTs and non-randomized controlled trials (nRCTs) that provide pre- and post-intervention quantitative data. Studies will be eligible if they involve human participants with stroke-induced gait impairments and evaluate the effects of mechanical vibration applied to the whole body or specific lower limb muscles. Studies including mixed neurological populations will be eligible only when data for participants with stroke can be separately extracted. Trials combining VT with other modalities (e.g., treadmill training) will be included only if the control group received the identical co-intervention without the active vibration component, ensuring the isolation of the vibration effect. We will exclude studies involving pediatric populations or those with other neurological comorbidities (e.g., Parkinson’s disease, multiple sclerosis) to prevent confounding factors. Additionally, studies utilizing non-mechanical vibration (e.g., acoustic vibration) or those lacking extractable quantitative data despite attempts to contact authors will be excluded.

### 2.4. Search Strategy

A comprehensive and systematic literature search will be conducted across major electronic databases, including PubMed, Embase, the Cochrane Library, Web of Science, and Scopus, from their inception through 31 July 2026. The search strategy will employ a robust combination of Medical Subject Headings and free-text keywords related to the condition (e.g., “Stroke,” OR “Hemiplegia,” OR “Cerebrovascular accident”), the intervention (e.g., “Vibration therapy,” OR “Whole-body vibration,” OR “Focal muscle vibration”), and gait-specific outcomes (e.g., “Gait speed,” OR “Walking capacity,” OR “Cadence,” OR “Step length”) (see Supplementary Table S1). To ensure a thorough retrieval of relevant evidence, we will also manually screen the reference lists of included articles and relevant systematic reviews. There will be no restrictions on the year of publication, but the language will be limited to English to ensure the accuracy of data interpretation.

### 2.5. Study Selection Process

All identified records will be imported into reference management software (e.g., EndNote) where duplicates will be removed. The selection process will follow a two-stage screening procedure conducted by two independent reviewers. In the first stage, titles and abstracts will be screened to exclude clearly irrelevant studies. In the second stage, the full texts of the remaining potentially eligible articles will be retrieved and assessed against the detailed inclusion and exclusion criteria. Any discrepancies regarding study eligibility will be resolved through discussion or consultation with a third independent reviewer to reach a consensus. The entire selection process will be documented and presented in a PRISMA 2020 flow diagram.

### 2.6. Data Extraction

Two reviewers will independently extract data using a standardized, pre-piloted form to ensure consistency and accuracy. Any disagreements between the two reviewers will be resolved through discussion and consensus; if consensus cannot be reached, a third reviewer will be consulted. Extracted information will include study characteristics (author, year, country), participant characteristics (age, sex, stroke type, stroke chronicity, lesion side, and baseline gait severity), and detailed intervention parameters. For studies in which VT is provided alongside rehabilitation, details of concomitant rehabilitation, including the type, frequency, and duration of treatment, will also be extracted when reported. Safety-related data, including adverse events, withdrawals related to the intervention, adherence, and reported contraindications, will also be extracted as secondary outcomes. Vibration and treatment characteristics will be extracted as distinct variables, including vibration frequency (Hz), amplitude/displacement (mm), acceleration when reported, session duration (min/session), treatment frequency (sessions/week), total number of sessions, total intervention period (days or weeks), posture, and anatomical application site. These parameters will be treated separately rather than combined into a single composite measure of vibration dose. Time since stroke onset will be extracted as reported in each study. For categorical analyses, three prespecified time-since-stroke categories will be used: ≤ 3 months after stroke onset (early phase), > 3 to ≤ 6 months after stroke onset (late subacute-to-early chronic phase), and >6 months after stroke onset (chronic phase), consistent with our previous meta-analytic framework for vibration therapy in stroke. Stroke type will be categorized as ischemic, hemorrhagic, mixed, or not reported. For studies including both ischemic and hemorrhagic stroke populations, stroke-type-specific data will be extracted when separately reported; otherwise, the study will be classified as mixed and will not be assigned to either stroke-type subgroup.

Crucially, to address the multidimensional nature of gait, outcome data will be extracted and categorized into the following four subgroups based on the specific metrics reported in the primary studies. To ensure consistent classification across studies, each eligible gait-related assessment will be assigned to a single prespecified analytical domain according to the outcome-to-domain taxonomy provided in Supplementary Table S2:

1. Gait Speed: This category will include measures reflecting the velocity of locomotion, such as the 10MWT (assessing both comfortable and fast speeds) and gait velocity (m/s).
2. Gait Endurance: This category will capture metrics of walking capacity over time or distance, primarily the 6MWT, which is a robust indicator of aerobic capacity and functional endurance in stroke survivors.
3. Functional Mobility: This domain will encompass tests that assess dynamic balance during movement and the ability to perform complex motor tasks, such as the TUG test and the Rivermead Mobility Index. The TUG will be interpreted as a composite measure of functional mobility that integrates walking, transfers, turning, and dynamic balance.
4. Spatiotemporal Parameters: This category will focus on the qualitative aspects of the gait cycle, including cadence (steps/min), step length (cm), stride length (cm), and temporal phase measures such as single/double support time.

To avoid unit-of-analysis errors when multiple correlated outcomes are reported from the same participant sample, a prespecified outcome-selection hierarchy will be applied within each domain. For gait speed, directly reported walking velocity will be prioritized, with comfortable or self-selected walking speed preferred over maximal or fast walking speed when multiple speed outcomes are available. For gait endurance, the 6MWT will be prioritized when multiple endurance measures are reported. For functional mobility, the TUG will be prioritized when multiple functional mobility measures are available. Spatiotemporal parameters, including cadence, step length, stride length, and support-phase measures, will be analyzed separately by metric rather than combined into a single pooled spatiotemporal effect. Within each meta-analysis, only one effect estimate from the same participant sample will be included for a given outcome construct and assessment time point.

Outcome assessment timing will also be extracted and categorized according to the timing of measurement relative to the intervention. For single-session studies, outcomes assessed within 24 h after the vibration session will be classified as immediate effects. For studies involving repeated treatment sessions, the primary post-intervention effect will be defined using the assessment performed closest to completion of the intervention. Follow-up assessments conducted after completion of the intervention will be analyzed separately and categorized as short-term follow-up (>0 to ≤3 months) or long-term follow-up (>3 months).

For outcomes where a lower value indicates better performance (e.g., time to complete the TUG or 10MWT), data will be standardized by inverting the values (multiplying by −1) to ensure that positive effect sizes consistently represent clinical improvement across all analyses.

### 2.7. Risk of Bias Assessment

The methodological quality of the included RCTs will be assessed using the Cochrane Risk of Bias 2 (RoB 2) tool, while non-randomized studies will be evaluated using the Risk of Bias In Non-randomized Studies of Interventions (ROBINS-I) tool. For RCTs, RoB 2 will assess bias arising from the randomization process, deviations from intended interventions, missing outcome data, measurement of the outcome, and selection of the reported result. For nRCTs, ROBINS-I will assess bias due to confounding, selection of participants, classification of interventions, deviations from intended interventions, missing data, measurement of outcomes, and selection of the reported result. For RCTs assessed using RoB 2, overall judgments will be categorized as low risk of bias, some concerns, or high risk of bias. For nRCTs assessed using ROBINS-I, judgments will be categorized as low, moderate, serious, or critical risk of bias, with no information assigned where appropriate. Discrepancies in the assessment will be resolved through consensus or consultation with a third reviewer.

### 2.8. Statistical Analysis

Quantitative data synthesis will be conducted using statistical software (e.g., Review Manager or R). To estimate the magnitude of the therapeutic effect, between-group differences in pre–post changes will primarily be used, and SMDs will be calculated as Hedges’ g with 95% confidence intervals, allowing for the integration of data from studies utilizing different measurement scales. When variance data are not directly reported, they will be derived from available study statistics where possible. Multi-arm and crossover trials will be handled using established meta-analytic procedures to avoid double counting of participants. To preserve statistical independence, only one effect estimate per participant sample will be included in a given meta-analysis for the same outcome construct and assessment time point, according to the prespecified outcome-selection hierarchy. Immediate, post-intervention, and follow-up effects will be synthesized separately according to the prespecified time-point definitions. When multiple assessments are reported within the same time window, only one assessment will be included in a given meta-analysis; the assessment closest to intervention completion will be selected for the primary post-intervention analysis, whereas the assessment closest to the end of the respective follow-up window will be selected for follow-up analyses. A random-effects model will be applied to account for the anticipated clinical and methodological diversity among studies. The primary efficacy analyses will be based on RCT evidence. RCTs and nRCTs will not be combined in the primary pooled analyses; instead, nRCTs will be synthesized separately as complementary evidence. When a sufficient number of clinically comparable nRCTs are available, a separate quantitative synthesis will be performed; otherwise, their findings will be summarized narratively. Statistical heterogeneity will be quantified using the I^2^ statistic and τ^2^, with prediction intervals reported where appropriate. I^2^ values of 25%, 50%, and 75% will be interpreted as low, moderate, and substantial heterogeneity, respectively, but heterogeneity will be interpreted in conjunction with clinical and methodological differences across studies.

To move beyond simple efficacy analysis and address the heterogeneity often seen in vibration studies, we will conduct prespecified meta-regression analyses to explore associations between effect estimates and key intervention parameters, specifically vibration frequency (Hz), amplitude/displacement (mm), and session duration (min/session). These parameters will be analyzed as distinct intervention characteristics rather than as interchangeable components of a composite vibration dose. Meta-regression will be conducted only when at least 10 independent studies are available for the moderator under consideration. Each moderator will initially be examined in a separate univariate meta-regression model to reduce the risk of overfitting. Multivariable meta-regression will be considered only when the available evidence is sufficient, with approximately 10 studies per covariate, and when the included moderators show adequate variation without substantial multicollinearity. If these criteria are not met, multivariable meta-regression will not be performed. Posture and anatomical application site will be considered as additional modality-specific moderators only when sufficient between-study variation is available and substantial multicollinearity with other intervention characteristics is absent. Given the mechanistic and biomechanical differences between WBV and FMV, the two modalities will be analyzed separately in the primary quantitative syntheses within each gait outcome domain. A pooled analysis combining WBV and FMV will not be used as the primary efficacy analysis and will be considered only as a secondary exploratory analysis when sufficient clinical and methodological comparability exists. Meta-regression analyses of vibration parameters will also preferentially be conducted within each vibration modality. Furthermore, subgroup analyses will be performed based on prespecified time-since-stroke categories (≤3 months, >3 to ≤6 months, and >6 months after stroke onset) and other prespecified clinical characteristics to explore potential sources of heterogeneity in treatment effects. Because the four gait domains represent distinct prespecified outcome constructs rather than repeated tests of the same endpoint, no formal multiplicity adjustment across these domains is planned. Given the multidimensional outcome framework and planned exploratory analyses, subgroup and meta-regression findings will be interpreted as exploratory and hypothesis-generating, with emphasis placed on effect estimates, 95% confidence intervals, and consistency of findings rather than on statistical significance alone. Sensitivity analyses will be conducted by sequentially excluding studies with a high risk of bias to verify the robustness of the pooled estimates. Publication bias will be assessed visually using funnel plots and statistically via Egger’s regression test when at least 10 studies are available for a meta-analysis. Funnel plot asymmetry will be interpreted cautiously, as it may reflect between-study heterogeneity or other small-study effects rather than publication bias alone. The certainty of evidence will be assessed using the GRADE approach for the principal outcomes within each gait domain and relevant comparison. Evidence from RCTs and nRCTs will be evaluated separately, and Summary of Findings tables will be prepared for the principal comparisons and outcomes where sufficient evidence is available.

## 3. Discussion

### 3.1. Rationale and Significance of the Study

Although VT has been increasingly applied in stroke rehabilitation, its effectiveness on gait recovery remains controversial, particularly when compared with more consistent effects observed for spasticity and balance outcomes [7,8,9,14,15,17]. Several RCTs have demonstrated reductions in lower-limb spasticity and improvements in balance indices following WBV; however, corresponding improvements in gait performance have been inconsistent or modest [13,14,15,17,18]. These findings suggest that gait may not respond uniformly to VT and that pooling outcomes without domain-specific stratification may obscure clinically meaningful effects.

Moreover, prior meta-analyses frequently combined heterogeneous gait-related outcomes or merged gait with balance measures, limiting clinical interpretability [14,15,17,19]. The present protocol therefore adopts a multidimensional gait framework to examine domain-specific effects and potential sources of heterogeneity [19,20,22,23,24].

### 3.2. Potential Mechanisms of Vibration Therapy

The primary mechanistic rationale for VT is that oscillatory mechanical stimuli activate muscle spindles and Ia afferents, thereby modulating spinal reflex excitability and influencing corticospinal drive [4,5,6]. Acute neuromuscular effects, including increased muscle activation and altered reflex responsiveness, have been demonstrated following WBV exposure [5,26]. However, their translation into clinically meaningful gait improvement appears to depend on intervention parameters and therapeutic context.

Clinical evidence suggests that these physiological effects do not translate uniformly into gait improvement. Acute or single-session WBV and some longer-term protocols have shown limited effects on gait performance, whereas repeated VT combined with task-oriented rehabilitation has produced more favorable functional outcomes [13,18,26,27]. Conversely, studies incorporating treadmill training or other functional contexts have reported improvements in walking speed, step length, and functional mobility [10,12,28,29]. Collectively, these findings suggest that the functional effects of VT may depend on both stimulation parameters and the context in which vibration is delivered.

Central mechanisms may also contribute to these effects. Neurophysiological evidence indicates that WBV can modulate activity in sensorimotor cortical regions associated with gait, alongside improvements in walking and functional mobility measures [30]. Thus, VT may influence both peripheral and central pathways relevant to gait recovery, although the magnitude and functional expression of these effects appear to vary across intervention protocols.

VT may also facilitate gait recovery through improved sensorimotor integration and proprioceptive reweighting. Repetitive afferent input may enhance proprioceptive information from the lower limbs and support temporal coordination and postural control during gait [3,12]. The preferential changes reported in some spatiotemporal measures rather than walking velocity further suggest that VT may influence gait stability and patterning differently from propulsive capacity [31]. This distinction reinforces the importance of domain-specific interpretation of gait outcomes.

### 3.3. Importance of Multidimensional Gait Outcome Categorization

A major limitation of previous syntheses is the aggregation of heterogeneous gait-related outcomes into single composite measures or their pooling with balance indices [14,15,17]. Gait, however, is a multidimensional construct comprising speed, endurance, spatiotemporal organization, and functional mobility, which reflect partially distinct aspects of locomotor performance [3,21,22]. Failure to distinguish these domains can obscure selective effects and inflate apparent inconsistency.

Recent trials support this domain-specific perspective, with VT-related changes varying across spatiotemporal parameters, walking speed, and functional mobility depending on the intervention protocol and therapeutic context [12,29,31,32]. Such differences may be obscured when heterogeneous gait measures are pooled into a single outcome.

These observations support the methodological decision in the present protocol to categorize gait outcomes into four domains, (1) gait speed, (2) gait endurance, (3) spatiotemporal parameters, and (4) functional mobility, thereby enabling more precise interpretation of VT effects and reducing outcome-related heterogeneity.

### 3.4. Addressing Parameter Heterogeneity via Meta-Regression

Substantial heterogeneity in VT parameters represents a central challenge in evidence synthesis. Across studies, vibration frequency, amplitude, intervention duration, posture, and co-interventions vary considerably [7,14,15,17,19]. Such variability may contribute to the small pooled effects and inconsistent findings reported in previous meta-analyses [14,15,17].

Controlled trials have reported divergent gait responses across different vibration parameters and intervention contexts, ranging from negligible effects with some stand-alone WBV protocols to improvements when vibration is integrated with task-oriented training [13,18,20,29]. Functional responses may also vary according to vibration intensity and the gait outcome assessed [21,32]. These findings support examining parameter heterogeneity as a potential contributor to variation in treatment effects rather than treating it solely as statistical noise. To systematically evaluate these relationships, the present review will employ meta-regression analyses using frequency, amplitude/displacement, and session duration as distinct intervention parameters. Given the differences in mechanical delivery and physiological targets between WBV and FMV, these associations will preferentially be examined within each vibration modality rather than across a single combined VT dataset. Analyses will also consider stroke stage and intervention context as potential sources of heterogeneity. This approach aims to transform heterogeneity from a limitation into an explanatory dimension, while avoiding assumptions of equivalence between mechanically distinct vibration modalities [17,20,32]. These moderator analyses will be considered exploratory and hypothesis-generating rather than confirmatory and are intended to inform, rather than define, future therapeutic protocols.

### 3.5. Clinical Translation and Safety Considerations

From a clinical perspective, VT offers practical advantages, including non-invasiveness and compatibility with existing rehabilitation workflows [5,6,7]. Most trials report good tolerability and no serious adverse events, although reporting of safety outcomes and contraindications remains inconsistent [7,19,32].

Clinical translation also depends on how VT is delivered. Positive gait outcomes have frequently been reported when vibration is integrated with supportive postures, visual feedback, or concurrent gait training, suggesting that therapeutic context may be as important as the vibration parameters themselves [10,12,29]. Localized vibration may also provide a targeted approach to gait-related impairments, although evidence remains limited [27].

Accordingly, the present synthesis will not only evaluate efficacy but also map reporting practices related to safety, adherence, and protocol implementation, with the goal of informing pragmatic recommendations for clinical use.

## 4. Limitations and Future Directions

This review is constrained by the quality and heterogeneity of available evidence. Many trials were underpowered, employed short intervention periods, or prioritized non-gait outcomes, limiting sensitivity to gait changes [14,15,17,19]. Additionally, variability in outcome definitions and follow-up durations restricts assessment of long-term effects [20,22]. The restriction to English-language publications may also introduce language-selection bias.

Future research should establish standardized reporting of VT parameters, adopt core gait outcome sets, and investigate stage-specific responses across different time-since-stroke categories [7,19,23]. Importantly, trials designed to test VT as an adjunct to task-oriented gait training may better elucidate its role in functional recovery [12,29].

## Data Availability

No new data were generated or analyzed in this protocol study. Data sharing is not applicable to this article.

## Supplementary Materials

The following supporting information can be downloaded at: https://www.mdpi.com/article/10.3390/biomedicines14092004/s1, Table S1: Database-specific search, Table S2: Prespecified taxonomy for assigning gait-related outcomes to analytical domains.

## Abbreviations

The following abbreviations are used in this manuscript:

VT	Vibration Therapy
WBV	Whole-Body Vibration
FMV	Focal Muscle Vibration
RCTs	Randomized Controlled Trials
nRCTs	Non-Randomized Controlled Trials
ROB	Risk of Bias
ROBINS-I	Risk of Bias In Non-randomized Studies of Interventions
PRISMA-P	Preferred Reporting Items for Systematic Review and Meta-Analysis Protocols
6MWT	6-Minute Walk Test
10MWT	10-Meter Walk Test
TUG	Timed Up and Go test
SMD	Standardized Mean Difference
GRADE	Grading of Recommendations Assessment, Development and Evaluation
