# Supplementary material for: Deciphering the Heterogeneity of Vibration Therapy for Post-Stroke Gait: A Systematic Review and Meta-Regression Protocol
Source: Biomedicines. 2026 Sep 6;14(9):2004. doi: 10.3390/biomedicines14092004 (PMC13604203; doi:10.3390/biomedicines14092004)
Supplement: Supplementary file 1 [file biomedicines-14-02004-s001.zip › biomedicines-4530850-supplementary.pdf]

Deciphering the Heterogeneity of Vibration Therapy for Post-Stroke Gait: A Systematic Review and Meta-Regression Protocol

Jeong-Woo Seo[1], Jin Mi Chun[1], Se-Ra Park[2], Ji-Woo Seok[1,*], Jung-Dae Kim[1,*]

[1] Digital Health Research Division, Korea Institute of Oriental Medicine, Daejeon 34054, South Korea

[2] Korean Medicine Convergence Research Division, Korea Institute of Oriental Medicine, Daejeon 34054, South Korea

***Correspondences:**

Jung-Dae Kim

Digital Health Research Division, Korea Institute of Oriental Medicine

1672 Yuseong-daero, Yuseong-gu, Daejeon 34054, Republic of Korea

Tel (office): +82-042-868-9415

E-mail: kjd@kiom.re.kr

Ji-Woo Seok

Digital Health Research Division, Korea Institute of Oriental

Medicine 1672 Yuseong-daero, Yuseong-gu, Daejeon 34054,

Republic of Korea Tel (office): +82-042-868-9734

E-mail: suk6124@kiom.re.kr

Supplementary Table S1. Database-specific search strategies

Database	Search strategy
**PubMed**	("Stroke"[MeSH Terms] OR stroke[tiab] OR "cerebrovascular accident"[tiab] OR hemiplegia[tiab]) AND ("Vibration"[MeSH Terms] OR "vibration therapy"[tiab] OR "whole body vibration"[tiab] OR "whole-body vibration"[tiab] OR WBV[tiab] OR "focal muscle vibration"[tiab] OR FMV[tiab]) AND ("Gait"[MeSH Terms] OR gait[tiab] OR walking[tiab] OR "walking speed"[tiab] OR ambulation[tiab] OR locomotion[tiab] OR "functional mobility"[tiab] OR "Timed Up and Go"[tiab] OR TUG[tiab] OR "6-minute walk test"[tiab] OR 6MWT[tiab] OR "10-meter walk test"[tiab] OR 10MWT[tiab]) AND ("randomized controlled trial"[Publication Type] OR "controlled clinical trial"[Publication Type] OR crossover[tiab] OR random*[tiab] OR trial[tiab] OR controlled[tiab] OR placebo[tiab])
**Embase**	('stroke'/exp OR stroke:ti,ab OR 'cerebrovascular accident':ti,ab OR hemiplegia:ti,ab) AND ('vibration'/exp OR 'vibration therapy':ti,ab OR 'whole body vibration':ti,ab OR 'whole-body vibration':ti,ab OR wbv:ti,ab OR 'focal muscle vibration':ti,ab OR fmv:ti,ab) AND ('gait'/exp OR gait:ti,ab OR walking:ti,ab OR 'walking speed':ti,ab OR ambulation:ti,ab OR locomotion:ti,ab OR 'functional mobility':ti,ab OR 'timed up and go':ti,ab OR tug:ti,ab OR '6 minute walk test':ti,ab OR 6mwt:ti,ab OR '10 meter walk test':ti,ab OR 10mwt:ti,ab) AND ('randomized controlled trial'/exp OR 'controlled clinical trial'/exp OR 'crossover procedure'/exp OR random*:ti,ab OR trial:ti,ab OR control*:ti,ab OR placebo:ti,ab)
**Web of Science**	TS=("stroke" OR "cerebrovascular accident" OR hemiplegia) AND TS=("vibration therapy" OR "whole-body vibration" OR "whole body vibration" OR WBV OR "focal muscle vibration" OR FMV) AND TS=(gait OR walking OR "walking speed" OR ambulation OR locomotion OR "functional mobility" OR "Timed Up and Go" OR TUG OR "6-minute walk test" OR 6MWT OR "10-meter walk test" OR 10MWT) AND TS=(random* OR trial OR controlled OR crossover OR placebo)
**Cochrane Library**	(MeSH descriptor: [Stroke] explode all trees OR stroke:ti,ab,kw OR "cerebrovascular accident":ti,ab,kw OR hemiplegia:ti,ab,kw) AND (MeSH descriptor: [Vibration] explode all trees OR "vibration therapy":ti,ab,kw OR "whole-body vibration":ti,ab,kw OR WBV:ti,ab,kw OR "focal muscle vibration":ti,ab,kw OR FMV:ti,ab,kw) AND (MeSH descriptor: [Gait] explode all trees OR gait:ti,ab,kw OR walking:ti,ab,kw OR "walking speed":ti,ab,kw OR ambulation:ti,ab,kw OR locomotion:ti,ab,kw OR "functional mobility":ti,ab,kw OR "Timed Up and Go":ti,ab,kw OR TUG:ti,ab,kw OR "6-minute walk test":ti,ab,kw OR 6MWT:ti,ab,kw OR "10-meter walk test":ti,ab,kw OR 10MWT:ti,ab,kw) AND

	(random*:ti,ab,kw OR trial:ti,ab,kw OR controlled:ti,ab,kw OR crossover:ti,ab,kw OR placebo:ti,ab,kw)
**Scopus**	TITLE-ABS-KEY(stroke OR "cerebrovascular accident" OR hemiplegia) AND TITLE-ABS-KEY("vibration therapy" OR "whole-body vibration" OR "whole body vibration" OR WBV OR "focal muscle vibration" OR FMV) AND TITLE-ABS-KEY(gait OR walking OR "walking speed" OR ambulation OR locomotion OR "functional mobility" OR "Timed Up and Go" OR TUG OR "6-minute walk test" OR 6MWT OR "10-meter walk test" OR 10MWT) AND TITLE-ABS-KEY(random* OR trial OR controlled OR crossover OR placebo)

The full search strategies developed for each database are presented. Detailed Boolean logic, database-specific field tags, controlled vocabulary, and search terms are provided to allow transparent replication of the search process.

Supplementary Table S2. Prespecified taxonomy for assigning gait-related outcomes to analytical domains

Gait-related assessment or metric	Prespecified analytical domain	Operational classification and selection rule
10-Meter Walk Test (10MWT)	Gait Speed	The 10MWT will be assigned exclusively to the gait-speed domain, regardless of whether the result is reported as walking velocity (m/s) or completion time (s). When both comfortable/self-selected and maximal/fast walking speeds are reported, comfortable/self-selected speed will be prioritized for the primary gait-speed analysis.
Directly reported gait/walking velocity	Gait Speed	Walking velocity expressed as distance per unit time (e.g., m/s) will be classified as gait speed. When several velocity measures are available from the same participant sample and time point, comfortable/self-selected velocity will be prioritized over maximal/fast velocity.
6-Minute Walk Test (6MWT)	Gait Endurance	The 6MWT will be assigned to the gait-endurance domain as a measure of sustained walking capacity. When multiple endurance measures are reported, the 6MWT will be prioritized.
Timed Up and Go (TUG)	Functional Mobility	The TUG will be assigned exclusively to the functional-mobility domain and interpreted as a composite measure incorporating walking, transfers, turning, and dynamic balance. It will not be additionally entered into the gait-speed domain. When multiple functional-mobility measures are reported, the TUG will be prioritized.
Rivermead Mobility Index	Functional Mobility	The Rivermead Mobility Index will be classified as a functional-mobility measure. If both the TUG and Rivermead Mobility Index are reported from the same sample and time point, the TUG will be prioritized according to the prespecified hierarchy.

Cadence	Spatiotemporal Parameters	Cadence will be analyzed as an individual spatiotemporal metric and will not be combined with other spatiotemporal parameters into a single composite effect.
Step length	Spatiotemporal Parameters	Step length will be analyzed separately as an individual spatiotemporal metric.
Stride length	Spatiotemporal Parameters	Stride length will be analyzed separately as an individual spatiotemporal metric.
Single-support time	Spatiotemporal Parameters	Single-support time will be analyzed separately as an individual temporal gait metric.
Double-support time	Spatiotemporal Parameters	Double-support time will be analyzed separately as an individual temporal gait metric.

Each eligible gait-related outcome will be assigned to one prespecified analytical domain only. When an assessment could plausibly reflect more than one construct, classification will follow this prespecified taxonomy rather than the terminology used by the original study. Within each meta-analysis, only one effect estimate from the same participant sample will be included for the same outcome construct and assessment time point.
